# All-Trans Retinoic Acid Induces CD4+CD25+FOXP3+ Regulatory T Cells by Increasing FOXP3 Demethylation in Systemic Sclerosis CD4+ T Cells

**DOI:** 10.1155/2018/8658156

**Published:** 2018-04-30

**Authors:** Xiaohong Sun, Yangfan Xiao, Zhuotong Zeng, Yaqian Shi, Bingsi Tang, Hai Long, Takuro Kanekura, Jiucun Wang, Haijing Wu, Ming Zhao, Qianjin Lu, Rong Xiao

**Affiliations:** ^1^Department of Dermatology, Second Xiangya Hospital, Central South University, 139 Ren-Min Road, Changsha 410011, China; ^2^Department of Dermatology, Xi'an Children's Hospital, Xi'an, Shaanxi 710003, China; ^3^Department of Dermatology, Kagoshima University Graduate School of Medical and Dental Sciences, 8-35-1 Sakuragaoka, Kagoshima 890-8520, Japan; ^4^Ministry of Education (MOE) Key Laboratory of Contemporary Anthropology and State Key Laboratory of Genetic Engineering, School of Life Sciences, Fudan University, Shanghai 200433, China; ^5^Hunan Key Laboratory of Medical Epigenomics, 139 Ren-Min Road, Changsha 410011, China

## Abstract

**Background:**

Retinoic acid (RA) is an active metabolite of vitamin A and has been reported to improve the clinical symptoms of patients with systemic sclerosis (SSc). However, the mechanism of RA in the prevention of SSc remains unclear. Regulatory T cells (Tregs) are a subpopulation of T cells with immunosuppressive activity. The quantitative and functional defects of Tregs may mediate the immune dysfunction in SSc. The addition of all-trans retinoic acid (ATRA) to human naïve CD4+ cells could promote the maturation of Tregs and increase the stable expression of Foxp3. In this study, we explored the role of RA on Tregs in SSc CD4+ T cells and its possible epigenetic mechanisms, so as to further understand the mechanisms of RA on SSc.

**Methods:**

CD4+ T cells were isolated from peripheral blood of SSc and treated with or without ATRA and/or transforming growth factor-*β* (TGF-*β*). The percentage of CD4+CD25+FOXP3+ Tregs was counted by flow cytometry. FOXP3 mRNA and protein levels were measured by quantitative real-time reverse transcriptase polymerase chain reaction and Western blotting, respectively. Bisulfite sequencing was performed to determine the methylation status of the FOXP3 proximal promoter sequences.

**Results:**

The expression of Tregs and FOXP3 in CD4+ T cells from patients with SSc increased in response to ATRA. Moreover, combined stimulation with ATRA and TGF-*β* resulted in the enhancement of these effects. Further studies revealed that stimulation with ATRA increased the expression of FOXP3 in SSc CD4+ T cells by downregulating FOXP3 promoter methylation levels.

**Conclusions:**

ATRA acts as an inducer of Treg response in SSc CD4+ T cells via demethylation of the FOXP3 promoter and activation of FOXP3 expression. This may be one of the molecular mechanisms for ATRA, and therefore, RA can be used for the treatment of SSc.

## 1. Introduction

Systemic sclerosis (SSc) is an autoimmune tissue disease characterized by the distinctive pathogenesis of microvascular dysfunction, diffused tissue fibrosis, and disturbances in the immune system [1]. Activated lymphocytes and autoantibodies are detected in SSc patients, which may be the main components of the abnormal active immune reactions that result in SSc [2]. Regulatory T cells (Tregs) are a subpopulation of CD4+ T cells with strong immunosuppressive activity, which has been reported to play important roles not only in the maintenance of self-tolerance but also in the negatively regulating immune responses against immune-mediated damage to the host [3]. Several studies have intensely focused on the phenotype and function of Tregs in SSc, and a majority of them have reported that quantitative reduction of Tregs may be an underlying cause of SSc [4, 5].

X chromosome-encoded transcription factor FOXP3 is a lineage-specifying factor responsible for the differentiation and suppressive function of Tregs [6]. Furthermore, recent studies have shown that it acts as a quantitative regulator of Tregs [7]. Epigenetic modifications are defined by the regulation of gene expression without changing the DNA sequence of the genome [8]. Recently, several groups have observed that the stability of FOXP3 expression correlates with DNA demethylation at its proximal promoter region [9]. *In vitro* treatment of CD4+CD25− cells with DNA hypomethylating agent 5-azacytidine or 5-aza-deoxycytidine (Aza) resulted in stable and increased FOXP3 expression in Tregs [10]. An increasing number of studies focus on changing the methylation status of DNA to induce stable FOXP3 expression and immunosuppressive function of Tregs [11–13].

All-trans-retinoic acid (ATRA) is an active and natural derivative of vitamin A, which has profound effects on cellular proliferation and differentiation. Moreover, it has been reported that ATRA exhibits both anti-inflammatory and immunoregulatory effects [14]. Recent studies have shown that FOXP3 expression and the immunosuppressive function of Tregs can be enhanced by ATRA in the immune system of both patients and mice [15, 16]. Therefore, ATRA has been considered single or adjuvant agents in the treatment of autoimmune diseases [17–19].

Retinoic acids (RA) have been reported to induce clinically significant therapeutic effects on SSc [20, 21]. However, the underlying molecular mechanism of RA in the prevention of SSc remains unclear. The previous studies reported that retinoids decreased collagen expression in the tight-skin mouse [22] and also in SSc human skin fibroblasts [23]. Tremendous progress has been made in understanding the immunological pathogenesis of SSc and the role of retinoic acid on Tregs. To further understand the immunoregulatory mechanism of RA, we explored the role of ATRA in Tregs in patients with SSc and its possible epigenetic mechanisms. We found that ATRA increased the proportion of Tregs and upregulated FOXP3 expression in SSc CD4+ T cells by inducing FOXP3 promoter demethylation. Together, our findings provide new evidence for the clinical use of retinoids in the treatment of SSc.

## 2. Materials and Methods

### 2.1. Subjects

Ten SSc patients were recruited from the Department of Dermatology at the Second Xiangya Hospital of Central South University between February 2013 and December 2014. Systemic sclerosis was diagnosed based on 2013 Classification Criteria for Systemic Sclerosis revised by American College of Rheumatology (ACR) and the European League against Rheumatism (EULAR) [24]. All the patients included in the study had typical characteristics of SSc, such as fibrosis of the skin, Raynaud's phenomenon, interstitial lung disease seen on high-resolution computed tomography, and SSc-related autoantibodies. And all of their clinical presentations have been scored according to the 2013 ACR/EULAR criteria. The disease manifestations are solely associated with SSc that means no participant has enough evidence of suffering from tumour, severe infection, or other internal organ failures unrelated to SSc. Meanwhile, other autoimmune diseases and overlapping connective tissue diseases have been also excluded from the analysis. Those who have been administrated with steroid and immunosuppressant within 3 months are also excluded. This study was approved by the Human Ethics Committee of the Second Xiangya Hospital of Central South University, and the methods were carried out in accordance with the approved guidelines. All the subjects enrolled in this study signed an informed consent form for their inclusion. The clinical information of SSc patients is provided in Table 1.

### 2.2. Isolation, Cell Culture, and Treatment of CD4+ T Cells

A total of 60 ml venous peripheral blood samples were drawn from each SSc patient and preserved with heparin. CD4+ T cells were isolated by positive selection using CD4 immunomagnetic beads, according to the manufacturer's protocol (Miltenyi, Germany), and cultured in RPMI 1640 medium (Gibco, USA). CD4+ T cells were then treated with or without 10 ng/ml TGF-*β* (Peprotech, USA) and/or 10 nm ATRA (Sigma, USA), in the presence of Dynabeads Human T-Activator CD3/CD28 (Life Technologies, USA) at a bead/T cell ration of 1 : 2 and 200 U IL-2 (Peprotech, USA) for 4 days.

### 2.3. Flow Cytometric Analysis

Isolated CD4+ T cell suspensions (1 × 10^5^ cells) were stained with antihuman monoclonal antibodies CD4-FITC and CD25-APC (BD Biosciences, USA), followed by fixation and permeabilization by a FOXP3 Fixation/Permeabilization kit (BD Biosciences, USA) and FOXP3-PE staining (BD Biosciences, USA) according to the manufacturer's instructions. Cells were then analyzed using a flow cytometer (FACS Canto II; BD Biosciences, San Jose, CA, USA). The percentage of CD4-, CD25-, and FOXP3-positive cells was calculated and analyzed using a FlowJo7.6.5 software (TreeStar Inc., USA).

### 2.4. RNA Isolation, cDNA Synthesis, and Real-Time Quantitative Reverse-Transcriptase Polymerase Chain Reaction (RT-PCR)

Total RNA was isolated from CD4+ T cells using the Trizol kit (Invitrogen, USA). cDNA synthesis was performed using the PrimeScript™ RT reagent Kit with a gDNA Eraser (Takara, Shiga, Japan). Levels of mRNA were quantified using the SYBR Premix Ex Taq™ real-time PCR Kit (Takara, Shiga, Japan). Real-time quantitative RT-PCR was performed using Rotor-Gene 3000 (Corbett Research, Sydney, Australia), and all samples were done in triplicate. FOXP3 mRNA levels were amplified by real-time quantitative RT-PCR using the following primers: CAAGTTCCACAACATGCGAC (forward) and ATTGAGTGTCCGCTGCTTCT (reverse). *β*-Actin was also amplified and used as an internal control to normalize the amount of total RNA using the following primers: GCACCACACCTTCTACAATGAGC (forward) and GGATAGCACAGCCTGGATAGCAAC (reverse) [5, 13].

### 2.5. Western Blotting

Total protein was extracted from the lysed CD4+ T cells. The protein concentration was then determined using the BCA™ Protein Assay Kit (Pierce Biotechnology, Rockford, USA). Proteins were separated by 12% SDS-polyacrylamide gel electrophoresis and then transferred onto a PVDF membrane (Millipore, USA). Membranes were blocked in TBST buffer containing 5% nonfat dry milk and then incubated overnight at 4°C with FOXP3 (1 : 1000; Cell signaling, USA) or *β*-actin (1 : 2000; Santa Cruz, USA) rabbit antihuman antibodies followed by incubation with the secondary mouse antirabbit antibody (1 : 5000, Santa Cruz, USA). The protein bands were detected using chemiluminescence and the gel imaging analysis system (ImageQuant LAS 4000, GE, USA). The relative expression levels were quantified using Quantity One software (Bio-Rad, USA).

### 2.6. Genomic DNA Extraction and Bisulfite Sequencing

Genomic DNA was isolated from CD4+ T cells using the TIANamp Genomic DNA kit (Tiangen Biotech, Beijing, China). Bisulfite conversion was performed using the EpiTect Bisulfite Kit (Qiagen, Germany). The 217 bp-long fragment of the FOXP3 promoter locus (−204 to +12) was amplified by the PCR assay using the designed primer sequences as follows: 5′-TATAATTAAGAAAAGGAGAAATATAGAGAG-3′ (forward) and 5′-TCAACCTAACTTATAAAAAACTATCAC-3′ (reverse). The amplified products were then cloned into the pGEM-T easy vector (Promega, USA). Ten independent clones from each subject were sequenced for each of the amplified fragments.

### 2.7. Statistical Analyses

Data were expressed as the mean ± SD. The results were statistically analyzed by one-way ANOVA or nonparametric Mann–Whitney *U* test for nonnormally distributed data among multigroups. All analyses were performed with SPSS 19.0 software (SPSS Inc., USA). Differences of *p* ≤ 0.05 were considered statistically significant.

## 3. Results

### 3.1. ATRA Treatment Increases the Percentage and Number of Tregs in SSc CD4+ T Cells

To investigate an optimum test concentration, we treated SSc peripheral blood CD4+ T cells with different concentrations of all-trans retinoic acid (0, 1, 10, and 100 nm ATRA) in our preliminary experiments. CD4+CD25+FOXP3+ Treg growth and numbers were evaluated using flow cytometry. The data indicated that maximum Treg growth and numbers were achieved at 10 nm ATRA concentration (date not shown). For the subsequent experiments, we therefore chose 10 nm ATRA as the test concentration.

**Figure 1:**
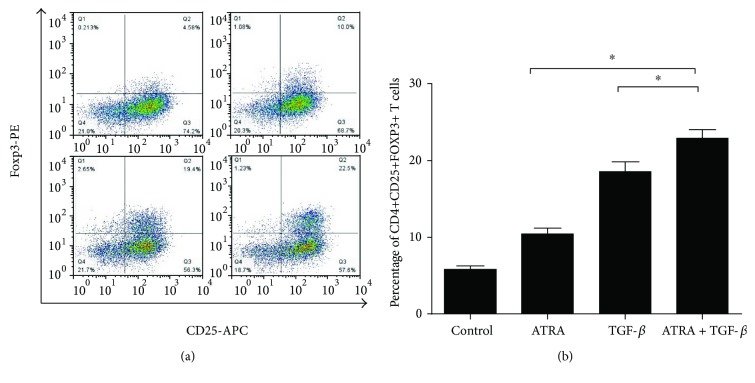
The percentage and number of Tregs in SSc CD4+ T cells. (a) SSc CD4+ T cells were treated with 10 nm ATRA and/or 10 ng/ml TGF-*β* in the presence of anti-CD3/CD28 beads and 200 U IL-2 for 4 days. CD4+CD25+FOXP3+ T cells were analyzed by flow cytometry. (b) The bar graph shows the percentage of CD4+CD25+FOXP3+ Tregs in samples of SSc in different groups. (b) The proportion of CD4+CD25+FOXP3+ Tregs was significantly increased in SSc CD4+ T cells treated with ATRA and/or TGF-*β* compared with the blank control group (all, *p* < 0.0.05). The ATRA and TGF-*β* combined stimulus group showed a significantly increased percentage of Tregs compared with the ATRA or TGF-*β* alone group (^∗^*p* < 0.05). Data are presented as the mean ± SD and are representative of at least 3 independent experiments.

To explore the therapeutic effect of the retinoid drug on SSc, CD4+ T cells were treated with TGF-*β* and/or 10 nm ATRA in the presence of anti-CD3/CD28 beads and IL-2 for 4 days *in vitro*. CD4+CD25+FOXP3+ Treg numbers were then assessed by flow cytometry. As shown in Figures 1(a) and 1(b), both the percentage and the absolute number of Tregs increased in SSc CD4+ T cells stimulated with ATRA alone or TGF-*β* alone compared with the blank control group. Furthermore, when SSc peripheral blood CD4+ T cells were jointly stimulated with ATRA and TGF-*β*, the percentage and number of Tregs in SSc CD4+ T cells increased significantly compared with the negative control or ATRA/TGF-*β* stimulus alone group.

### 3.2. ATRA Increases mRNA and Protein Levels of FOXP3 in SSc CD4+ T Cells

**Figure 2:**
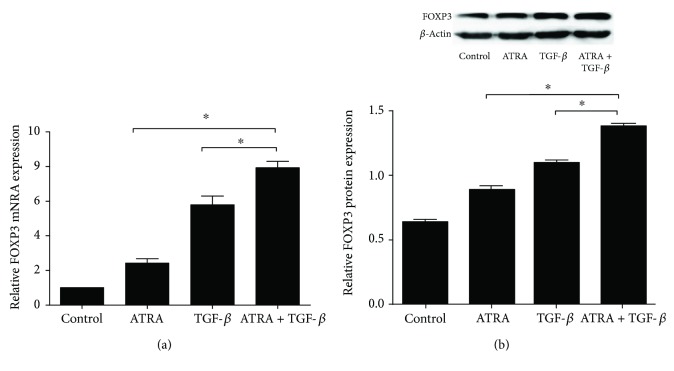
The expression of FOXP3 in SSc CD4+ T cells. FOXP3 mRNA and protein levels were analyzed by real-time quantitative PCR and Western blotting, respectively, in different cell groups. (a) The bar chart shows relative FOXP3 mRNA expression in ATRA-treated SSc CD4+ T cells normalized to *β*-actin. (b) The image shows the protein expression of FOXP3 and *β*-actin on the Western blots. The bar chart shows quantification of the Western blot band intensities of FOXP3 protein levels normalized to *β*-actin. FOXP3 mRNA and protein levels were increased in SSc CD4+ T cells stimulated with ATRA and/or TGF-*β* compared with the blank control group (all, *p* < 0.05). The ATRA and TGF-*β* combined stimulus group showed a significantly increased percentage of Tregs compared with the ATRA or TGF-*β* alone group (^∗^*p* < 0.05). Data are presented as the mean ± SD and are representative of at least 3 independent experiments.

FOXP3, a specific molecular marker for CD4+CD25+FOXP3+ Tregs, is required for the development and function of Tregs [6]. In order to study how ATRA regulates the induction of Tregs, we focused on the expression of FOXP3 in ATRA-treated cells. FOXP3 mRNA and protein levels were increased in SSc CD4+ T cells stimulated with ATRA alone or TGF-*β* alone compared with control-treated cells (*p* < 0.05, Figures 2(a) and 2(b)). In addition, we also found significantly upregulated levels of FOXP3 mRNA and protein when SSc CD4+ T cells were jointly stimulated with ATRA and TGF-*β* compared with the negative control or ATRA/TGF-*β* stimulus alone group. These results are consistent with the effects of ATRA on Treg amplification shown above.

### 3.3. ATRA Decreases DNA Methylation Levels at the FOXP3 Promoter in SSc CD4+ T Cells

**Figure 3:**
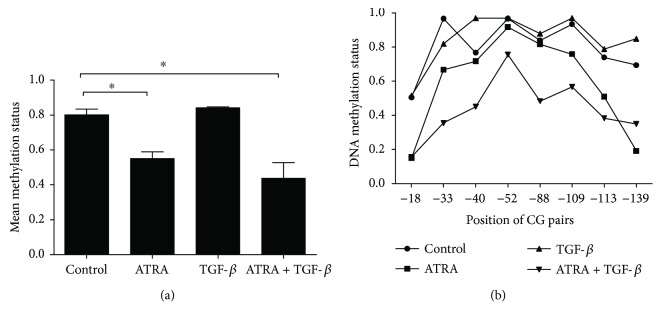
DNA methylation levels at the FOXP3 promoter in SSc CD4+ T cells. The average methylation level of the FOXP3 gene promoter sequence was cloned and sequenced as described in Materials and Methods. (a) The bar graph shows the mean DNA methylation status of the FOXP3 promoter in different groups. (b) The broken line shows the mean methylation status of 8 CG pairs in the promoter region of FOXP3 within 10 sequenced clones in different groups. The DNA methylation status of the FOXP3 promoter region was significantly decreased in SSc CD4+ T cells with the stimulation of the ATRA alone group or the ATRA and TGF-*β* combined group compared with negative controls (^∗^*p* < 0.05). The mean methylation level was not obviously different in the TGF-*β* alone group (*p* > 0.05). Moreover, no significant changes in the promoter methylation level of FOXP3 were observed in the ATRA alone group compared with the ATRA and TGF-*β* combined group. (*p* > 0.05). Data are presented as the mean ± SD and are representative of at least 3 independent experiments.

DNA methylation is an important part of epigenetics and has been shown to be involved in the regulation of FOXP3 expression [9–11]. We investigated whether ATRA treatment affected the DNA methylation status of the FOXP3 gene in SSc CD4+ T cells. The methylation status of a 217 bp (−204 bp to +12 bp) fragment in the FOXP3 promoter sequence was analyzed by bisulfite sequencing. As shown in Figures 3(a) and 3(b), the average methylation level of the 8 CG pairs was significantly downregulated in SSc CD4+ T cells stimulated with the ATRA alone group or the ATRA and TGF-*β* combined group compared with the negative control. However, the mean methylation level in the TGF-*β* alone group was not significantly different compared with the negative control. Moreover, no significant changes in the promoter methylation level of FOXP3 were observed in the ATRA alone group in comparison with the ATRA and TGF-*β* combined group.

## 4. Discussion

The immunological aspects and multiple altered immunological processes are closely related to the development and severity of the SSc [2]. Tregs possess immunosuppressive capabilities and play an essential role in the maintenance of self-tolerance and preventing severe autoimmune reactions deleterious to the host [3, 25]. The forkhead-box transcription factor FOXP3 has been identified as the master regulator of Treg differentiation, function, and survival [6]. Dysfunctional Tregs or reduced Treg numbers act as a central factor in the development of various autoimmune disorders. Moreover, the absence or mutations of FOXP3 result in immune dysregulation and multiorgan autoimmunity [26, 27]. Both Tregs and a specific molecular marker FOXP3 significantly influence the pathogenesis and development of SSc as well as its potential therapeutic targets [4, 5].

ATRA has been reported to control and modulate the autoimmune diseases and has a clinically significant therapeutic effect on SSc [20, 21]. In this study, we treated SSc CD4+ T cells with or without TGF-*β* and/or ATRA *in vitro*. The analysis indicated that the proportion of CD4+CD25+FOXP3+ Tregs and FOXP3 expression were significantly increased in SSc CD4+ T cells after ATRA alone treatment or ATRA and TGF-*β* combined treatment. It is indicated that ATRA can expand the number of Tregs via the activation of the FOXP3 expression in SSc CD4+ T cells, and this role was further enhanced by combined treatment with TGF-*β*. These findings highlight a novel pharmacological mechanism of action for ATRA in the treatment of SSc. However, some studies have shown that RA enhanced the proportion of FOXP3+ cells in TGF-*β*-containing cultures, but failed to promote the expansion of FOXP3+CD4+Tregs alone. In our study, ATRA not only can enhance the expression of TGF-*β* induced FOXP3 but also can expand the number of Tregs and the expression of FOXP3 alone in SSc CD4+ T cells. We speculate that SSc is a complex autoimmune disease; the environmental events and genetic predisposition have great changes and disorder in the progression and development of the disease. Therefore, these changes may cause the SSc patients to become more sensitive to RA. Induced Tregs and FOXP3 expression after treatment with ATRA alone have been reported in another autoimmune disease like type 1 diabetes [28]. However, further research is needed to validate this speculation and to understand the immunological treatment mechanism of RA.

Epigenetic modifications are crucial for transcription and expression of FOXP3. A completely demethylated FOXP3 promoter region is involved in stabilizing FOXP3 expression and for a committed Treg phenotype in humans. This may provide future implications for expanding Tregs in immunotherapy. To test whether ATRA can influence the methylation level of the FOXP3 promoter region, we chose a conserved fragment (−204 to +12) on the FOXP3 promoter region, which includes 8 CG pairs to analyze the DNA methylation status. Our data showed that the average methylation level of the eight 8 pairs at this promoter region was decreased after ATRA alone treatment or ATRA and TGF-*β* combined treatment in SSc CD4+ T cells. These observations may elucidate the effect of ATRA in reducing DNA methylation, especially at the proximal promoter in the FOXP3 locus, which plays an important pharmacological role in the treatment of SSc, and this function is not affected by TGF-*β*. Moreover, the mean methylation level in this FOXP3 promoter region did not change significantly with TGF-*β* treatment, suggesting that FOXP3 promoter methylation is not a target role in the TGF-*β*. These results are in line with a previous study that showed no obvious additional demethylation at the human FOXP3 promoter when TGF-*β* was added during cell culture [9]. Currently, very little is known about the therapeutic mechanisms of ATRA in the treatment of SSc. Our results have demonstrated that ATRA treatment effectively increased the proportion of Tregs in SSc CD4+ T cells by downregulating the methylation levels of the FOXP3 promoter region and subsequently activating and enhancing FOXP3 expression. Overall, our findings provide novel insights into understanding the pharmacological effects of ATRA in the treatment of SSc.

## Figures and Tables

**Table 1:** Clinical summary of SSc patients.

Patient	Sex/age	ACR/EULAR score^a^	Medications^b^
1	F/61	12	None
2	F/51	11	None
3	F/58	17	None
4	F/41	14	None
5	F/44	12	None
6	F/56	16	None
7	F/54	11	None
8	F/52	15	None
9	F/42	18	None
10	F/60	14	None

^a^According to 2013 Classification Criteria for SSc. ^b^Whether SSc patients had steroid and immunosuppressant within three months when blood was collected for the experiments.
